# Serial lumbar puncture reduces cerebrospinal fluid (CSF) infection during removal of hemorrhagic CSF in aneurysmal subarachnoid hemorrhage after endovascular coiling

**DOI:** 10.7555/JBR.32.20170028

**Published:** 2018-01-17

**Authors:** Chen Liang, Ling Yang, Shiwen Guo

**Affiliations:** 1. Department of Neurosurgery, the First Affiliated Hospital of Xi'an Jiaotong University, Xi'an, Shaanxi 710061, China; 2. Department of Aeromedical Physical Examination, Xi'an Civil Aviation Hospital, Xi'an, Shaanxi 710082, China.

**Keywords:** serial lumbar puncture, cerebrospinal fluid infection, aneurysmal subarachnoid hemorrhage

## Abstract

The present study aimed to compare the complications and clinical outcomes of serial lumbar puncture (LP) and lumbar cerebrospinal fluid (CSF) drainage (LD) of patients with aneurysmal subarachnoid hemorrhage and provide more evidence to guide clinical management. In this retrospective study, 41 and 39 aneurysmal subarachnoid hemorrhage patients were enrolled in the LP and LD group, respectively. Clinical outcomes, including CSF infection, intracerebral hemorrhage, vasospasm, hydrocephalus, death, length of stay, duration of drainage and the Glasgow Outcome Scale score were compared between the two groups. By comparing with the LP group, the LD group showed a significantly higher rate of CSF infection (*P=*0.029) and shorter duration of drainage (*P*<0.001). Both groups displayed similar rates of vasospasm, hydrocephalus, intracerebral hemorrhage, the Glasgow Outcome Scale score one month after endovascular coiling and length of stay (*P>*0.05, respectively). In conclusion, both LD and serial LP are effective methods in the treatment of aneurysmal subarachnoid hemorrhage; besides, serial LP can reduce the incidence of CSF infection in draining hemorrhagic CSF in aneurysmal subarachnoid hemorrhage after endovascular coiling.

## Introduction

As a disease needing neurosurgery, aneurysmal subarachnoid hemorrhage (aSAH) is characterized with high morbidity and mortality. Although improved diagnostic techniques, emergency treatments and operations have lowered the mortality rate of aSAH, the overall outcome is still undermined by some complications, such as vasospasm and hydrocephalus^[1]^. The pathophysiology of aSAH is not completely known, while, undoubtedly, the presence of blood in the subarachnoid space is one important factor associated with these complications^[2]^. Removal of bloody cerebrospinal fluid (CSF) plays an important role in the management of aSAH. Various studies have revealed that early removal of hemorrhagic CSF after the onset of aSAH can significantly cut down the incidence of vasospasm and hydrocephalus associated with aSAH^[3^‒^4]^.

There exist three primary methods to drain hemorrhagic CSF: serial lumbar puncture (LP), lumbar CSF drainage (LD) and external ventricular drainage. External ventricular drainage, a standard approach in treating SAH-associated hydrocephalus, has been proven to be effective in CSF drainage^[1]^, but it needs special surgical instruments and should be performed in the operating room, generally for patients with massive intraventricular hemorrhage or acute hydrocephalus. Even so, both serial LP and LD have been considered as conventional CSF drainage methods in the clinical treatment of aSAH and widely applied around the world. Unlike external ventricular drainage, serial LP and LD can be performed rapidly at bedside without the risk of sedation or general anesthesia.

As a classical neurosurgery, serial LP has remained a basic method in clinical treatment of aSAH. Many studies have confirmed that draining hemorrhagic CSF by serial LP can significantly depress the complications of aSAH and improve the outcome of patients with aSAH^[5^‒^7]^. Recently, LD has gradually become another option to promote the clearance of blood from the subarachnoid space. Clinical studies have confirmed that LD is also an effective way to reduce aSAH associated incidence of vasospasm and hydrocephalus^[3^,^7]^.

However, each CSF drainage method has its advantages and disadvantages and comparisons between these methods are rare. As the two most common methods, it is necessary to compare the safety and efficacy of serial LP and LD in treating aSAH. Therefore, this study aimed to compare the complications and clinical outcomes of serial LP and LD of patients with aSAH and discover more evidence to guide management.

## Patients and methods

### Patients

A total of 80 patients with aSAH were included in this retrospective study. All the patients underwent endovascular coiling of intracranial aneurysm at the Department of Neurosurgery of the First Affiliated Hospital of Xi’an Jiaotong University between January 2009 and January 2014. The inclusion criteria were (1) spontaneous primary aSAH confirmed by CT scan and digital subtraction angiography (DSA); (2) Hunt & Hess grades Ⅱ to Ⅳ; (3) Fisher grades 2, 3, 4; (4) receiving the endovascular coiling within 36 hours after admission and receiving hemorrhagic CSF drainage less than 48 hours after onset. According to the different methods of hemorrhagic CSF drainage in the postoperative period, patients were divided into two groups. One group underwent serial LP, and the other LD. All other aspects of treatment were similar. This study was approved by the ethical committee of the First Affiliated Hospital of Xi’an Jiaotong University. Patient consent was not required because of the retrospective nature of the study.

**Tab.1:** Characteristics for patients in both groups

Characteristics	LP	LD	*P* value
No. of patients	41	39	0.532
Mean age (years)	55.95±10.60	55.5±11.52	
Gender [*n*(%)]			0.267
Male	11(26.83)	15(38.34)	
Female	30(73.17)	24(61.54)	
Hunt & Hess grade [*n*(%)]			0.898
II	12(29.27)	10(25.64)	
III	20(48.78)	21(53.85)	
IV	9(21.95)	8(20.51)	
Fisher grade [*n*(%)]			0.374
2	4(9.76)	7(17.95)	
3	28(68.29)	21(53.85)	
4	9(21.95)	11(28.20)	
Aneurysm type [*n*(%)]			0.890
Anterior circulation	30(73.17)	28(71.79)	
Posterior circulation	11(26.83%)	11(28.21%)	

### Clinical management

According to a consistent protocol with the exception of different CSF drainage management methods, all patients were given similar treatments. The diagnosis of aneurysm was confirmed by DSA and the initial CT scan was adopted to confirm and classify SAH using a Fisher grading system. Patients with ruptured aneurysm received early endovascular coiling within 36 hours after admission. Other treatments included blood pressure control, administration of nimodipine, maintenance of euvolemia and normal circulating blood volume, daily transcranial Doppler examination, rigorous monitoring and correction of electrolytes, blood gases and intracranial pressure (ICP), regular CSF examination, and CT scan.

Serial lumbar puncture was performed daily from postoperative day 1 with the following steps. The patient was placed in the lateral position. The puncture site was L4‒L5 or L3‒L4 intervertebral space. After regular skin disinfection, 1% lidocaine solution was injected from the skin into the interspinous ligament for local infiltrative anesthesia. A lumbar puncture needle was inserted into the subarachnoid space and hemorrhagic CSF was drained. Each time, a maximum of approximately 20 mL CSF was slowly drained; and the ICP was maintained with approximately 15 cmH_2_O.

Lumbar CSF drainage was also performed since postoperative day 1. The procedure of lumbar CSF drainage was similar to that of lumbar puncture as described above. After the needle was inserted, a catheter was put through the needle into the subarachnoid space, and then the needle was removed. The catheter was then fixed to the skin and connected to an external drainage system. The collection rate was targeted at 5 to 10 mL per hour. CSF drainage in each group was stopped once the CSF was no longer visibly hemorrhagic.

### Outcome measurements

#### CSF infection

CSF was collected and analyzed every day. If leukocytosis in CSF was observed, CSF cultivation was performed; once positive, CSF infection was confirmed. Abnormal CSF findings (CSF/blood glucose ratio less than 0.5 and a neutrophilic CSF pleocytosis) and clinical manifestations (body temperature>38 °C) with negative CSF culture were defined as suspected infection. Both situations were defined as CSF infection.

#### Intracerebral hemorrhage

Regular CT scan was performed per 2 or 3 days. Additionally, patients received emergency CT scan anytime if neurological symptoms worsened or new symptoms appeared. Intracerebral hemorrhage was confirmed by CT scans.

#### Vasospasm

Daily transcranial Doppler was performed in all patients; if the mean arterial velocity was higher than 120 cm/s, they were suspected of vasospasm^[8]^. Some patients with normal results by transcranial Doppler were also suspected of vasospasm if CT scans revealed a new region of cerebral infarction consistent with the distribution area of the intracranial artery. All patients underwent cerebral angiography when vasospasm was suspected on clinical evidence.

#### Hydrocephalus

The diagnosis of hydrocephalus was based on neuro-imaging examinations. If CT scan showed that the dilated lateral ventricles and the third ventricle and the cella media index were above 0.25, and the third ventricle width was larger than 7 mm, hydrocephalus was confirmed.

#### Glasgow outcome scale (GOS) score

To evaluate the short term outcome, the GOS score was measured one month after endovascular coiling.

#### Length of stay and duration of drainage

The analysis of duration of drainage excluded those patients who suffered CSF infection, for they needed an extended period of lumbar CSF drainage. In addition, patients who died were excluded from analysis of length of stay and duration of drainage.

### Statistical analysis

Data were analyzed using SPSS 17.0. Chi-square test, Fisher’s exact test and Yates’ continuity correction test were applied to compare categorical variables between groups. Student *t* test was used to compare continuous data, which were presented as mean±standard deviation. *P<*0.05 was considered as statistically significant.

## Results

### Patient demographic and baseline characteristics

Patient characteristics, including age, gender, Hunt & Hess grade, Fisher grade, and aneurysm type in the two groups, are shown in ***Table 1***. A total of 80 patients with aSAH were included in this study with 41 patients in the LP group and 39 in the serial LD group. Patient characteristics had no statistically significant differences between the two groups, including age, gender, Hunt & Hess grade, Fisher grade and aneurysm type (*P>*0.05, respectively).

### CSF infection

As shown in ***Table 2***, 6 patients had CSF infection during the removal of hemorrhagic CSF. All of them received lumbar CSF drainage (LD). Four of them were confirmed by CSF culture and two were suspected of infection. Two of these patients died of severe infection. No patient had CSF infection in the serial LP group. The CSF infection rate in the LD group was significant higher than that of the LP group (*P=*0.029).

**Tab.2:** Outcomes for patients in both groups

Outcome	LP	LD	*P* value
CSF infection [*n*(%)]	0(0)	6(15.38)	0.029
Intracerebral hemorrhage [*n*(%)]	0(0)	1(2.56)	0.487
Vasospasm [*n*(%)]	7(17.07)	6(15.38)	0.838
Hydrocephalus [*n*(%)]	2(4.88)	0(0)	0.494
Death [*n*(%)]	0(0)	3(7.69)	0.222
Length of stay (LOS)	18.75±7.03	20.65±11.49	0.532
Duration of drainage	6.17±1.20	4.67±0.77	0.000
GOS score [*n*(%)]			
1(%)	0(0)	3(7.69)	0.287
2(%)	0(0)	0(0)	
3(%)	6(14.63)	5(12.82)	
4(%)	8(19.51)	5(12.82)	
5(%)	27(65.85%)	26(66.67%)	

CSF: cerebrospinal fluid; GOS: Glasgow outcome scale.

### Intracerebral hemorrhage

A patient in the LD group developed intracerebral hemorrhage and died of cerebral hernia. The rate of intracerebral hemorrhage in the LD group was 2.56%. No patient developed intracerebral hemorrhage in the serial LP group. Although there was a slight decrease in the risk of intracerebral hemorrhage with serial LP, no statistically significant difference was found (*P=*0.487).

### Vasospasm

Thirteen patients had vasospasm, 7 in the serial LP group (17.07%) and 6 in the LD group (15.38%). Although the LD group had a lower rate of vasospasm, there was no statistically significant difference between the two groups (*P=*0.838).

### Hydrocephalus

Two patients in the serial LP group developed hydrocephalus with a rate of 4.88%. No patient developed hydrocephalus in the LD group. There was no statistically significant difference between the two groups (*P=*0.494).

### Death

As presented above, 3 patients died in the LD group. Two died of severe infection. One died of intracerebral hemorrhage, but there was no death in the serial LP group. The mortality showed no statistically significant difference between the two groups (*P=*0.222).

### Length of stay and duration of drainage

No significant difference was observed in the length of stay between the two groups (*P=*0.532), but the duration of drainage in LD group was significantly shorter than that of the serial LP group (4.67±0.77 *vs*. 6.17±1.20; *P=*0.000).

### GOS score

All patients were assessed with GOS scores 30 days after endovascular coiling. No significant differences in GOS scores between the two groups were found (*P=*0.287).

## Discussion

Despite advances in treatment of aSAH, the mortality rate of the disease remains high worldwide. The median mortality rate is 27% to 44% according to different epidemiological studies^[9]^. Even so, 8% to 20% of those who survived suffered permanent disability^[9]^.

The management of aSAH is a complex procedure, and the removal of hemorrhagic CSF plays an important role in this procedure. Lumbar CSF drainage for the treatment of aSAH-associated complications such as hydrocephalus and vasospasm has been reported to be safe and effective, recommended by some guidelines^[1]^. Howe ver, lumbar CSF drainage could also induce severe fatal complications, such as infections^[10]^ and intracerebral hemorrhage^[11]^, and the overall complication rate reached 44.4%^[12]^. As a classical method, serial lumbar puncture has always been used in the clinical treatment of aSAH and described as safe and effective^[5^‒^7]^. However, to our knowledge, the comparison of safety and efficacy between these two methods in the treatment of aSAH has not been reported.

LD associated intracerebral hemorrhage is usually related to excessive CSF drainage^[13]^. In the study, one patient in the LD group developed intracerebral hemorrhage and died of cerebral hernia in spite of collection rate control of CSF, which may indicate that the CSF drainage plan should be made individually by the ICP and blood volume. This should be investigated further. However, the present study also found that lumbar CSF drainage presented no increased risk of intracerebral hemorrhage when compared with serial lumbar puncture. Both methods were safe.

Prevention and management of LD associated CSF infection pose a major challenge. The CSF infection rate of LD was 5%‒18% according to different studies^[12^,^14^‒^16]^. In the present study, 6 patients in the LD group (15.38%) suffered from CSF infection, 4 of whom (10.26%) were confirmed by CSF culture and the rate was similar to that reported previously. Some researchers considered LD as a significant risk factor for meningo-ventriculitis^[17]^. As the risk of LD associated CSF infection was complicated, the patients with long term duration of catheter placement (more than 4 days) or with puncture site leakage are at greater risk^[18]^. Other risk factors, including infection that originated from insertion procedure, the frequency of catheter manipulation and sampling, the presence of blood in the CSF, have also been identified^[19]^. In contrast, our study demonstrated that serial lumbar puncture showed a lower infection rate during drainage of hemorrhagic CSF, which is consistent with previous report^[7]^. The reasons may be related to the shorter duration of needle placement in each insertion procedure and the lower rate of puncture site leakage. LD related CSF infection is a preventable disease. Leverstein-van *et al.* have reported that they decreased the proportion of probable drain-related infection from 37% to 9% by strict adherence to accurately defined procedures, including improved awareness, focusing on the standard operating procedures, the diagnostic and therapeutic algorithm, timely administration of prophylaxis, and the improvement of the drainage system^[20]^.

In the present study, both LD and serial LP showed similar efficacy in the treatment of aSAH related vasospasm and hydrocephalus. Although there was no statistically significant difference, the LD group exhibited lower vasospasm and hydrocephalus rates. Meanwhile, even though GOS score and LOS in the two groups had no statistically significant difference, the LD group had significantly shorter drainage duration than the serial LP group. These results suggest that the main outcomes in the treatment of aSAH between the two groups are the same and lumbar CSF drainage seem to be more effective than the serial lumbar puncture.

There are several limitations in this study. First of all, the selection bias is a potential problem in a retrospective non-randomized study. Secondly, the long term outcomes of these patients were not evaluated due to the loss of follow-up. Thirdly, patients with severe SAH were not enrolled in this study. Finally, this study is a single-center design with a long time span and only a small number of patients were included, which may affect the results. Therefore, the results of the present study still need randomized control trials to confirm.

In conclusion, both lumbar CSF drainage and serial lumbar puncture are effective methods in the treatment of aSAH. Serial lumbar puncture reduces the CSF infection during the drainage of hemorrhagic CSF in aSAH after endovascular coiling.
